# A rare Ewing-like small round cell tumor in prostate: a case report and literature review

**DOI:** 10.1007/s00432-023-05585-2

**Published:** 2024-03-01

**Authors:** Zhen Wang, Jian Ye, Junjie Hu, Nan Zhang, Yichu Yuan

**Affiliations:** 1 Key Laboratory of Tumor Microenvironment and Immune Therapy of Zhejiang Province, Second Affiliated Hospital, Zhejiang University School of Medicine, Hangzhou, 310009 China; 2 Department of Breast Surgery, Second Affiliated Hospital, Zhejiang University School of Medicine, Hangzhou, 310009 China; 3 Department of Surgery, Songyang People’s Hospital, Lishui, 323700 China; 4 Department of Urology, Lanxi People’s Hospital, Jinhua, 321100 China; 5 Department of Urology, Second Affiliated Hospital, Zhejiang University School of Medicine, No.88 Jiefang Road, Hangzhou, 310009 China

**Keywords:** Small round cell tumor, Ewing’s sarcoma, Prostate, Pathological examination, Whole-genome sequencing

## Abstract

**Background:**

Small round cell tumor (SRCT) is a group of malignancy with similar optical microscopic morphology. Despite its low incidence, SRCT has a high malignant degree and poor prognosis. Besides, atypical clinical symptoms make it difficult in preoperative diagnosis.

**Case report:**

A 67-year-old man was presented to the outpatient service with dysuria and weak urine stream lasting for 3 months. After oral treatment with tamsulosin and finasteride for 2 months, the symptoms worsen. Transurethral prostate holmium laser enucleation was operated and postoperative pathology result revealed small blue round cell malignant tumor. Further immunohistochemistry and fluorescence in situ hybridization examination indicated Ewing-like SRCT. So a Da Vinci Robotic prostatectomy was performed further and whole-genome sequencing was conducted. Several gene mutations including *RAF1, ARID1A, SMARCA4,* and *BCL2L11* were found but no FDA-approved drug could treat specifically. Then the patient received Ewing-type therapeutic regimens treatment and has been followed up to date (over 24 months).

**Conclusion:**

Because of its non-elevated serum PSA level, prostate SRCT is often ignored as a possibility of malignant tumor and regarded as benign prostatic hyperplasia (BPH). The possibility of prostate SRCT need to be considered if dysuria symptoms could not alleviate significantly after a period of oral treatment.

## Introduction

Small round cell tumor (SRCT) is a heterogeneous tumor composed of relatively small undifferentiated cells that are round or oval in shape and densely arranged. It has less cytoplasm, round nuclei, uniform distribution, rough chromatin, and small or inconspicuous nucleoli under light microscopy (Rajwanshi et al. 2009). Despite having similar cell morphology under microscope, the pathological entities of SRCT may come from extremely different lineages, including (1) epithelial tumors, such as small cell carcinoma; (2) mesenchymal tumors, including malignant solid tumors in children and other small circles cell sarcoma; (3) tumors with overlapping features such as lymphoma and melanoma. We now report a rare Ewing-like small round cell tumor that occurs in the prostate with unique genetic phenotype.

## Case presentation

A 67-year-old man was presented to his local hospital outpatient service with dysuria and weak urine stream for 3 months. Prostate ultrasound indicated benign prostatic hyperplasia (BPH) and no obvious nodule was detected. Besides, the level of serum prostate-specific antigen (PSA) was not elevated. So he was given oral treatment with tamsulosin and finasteride for 2 months, but he felt the dysuria symptoms gradually worsened.

**Fig. 1:**
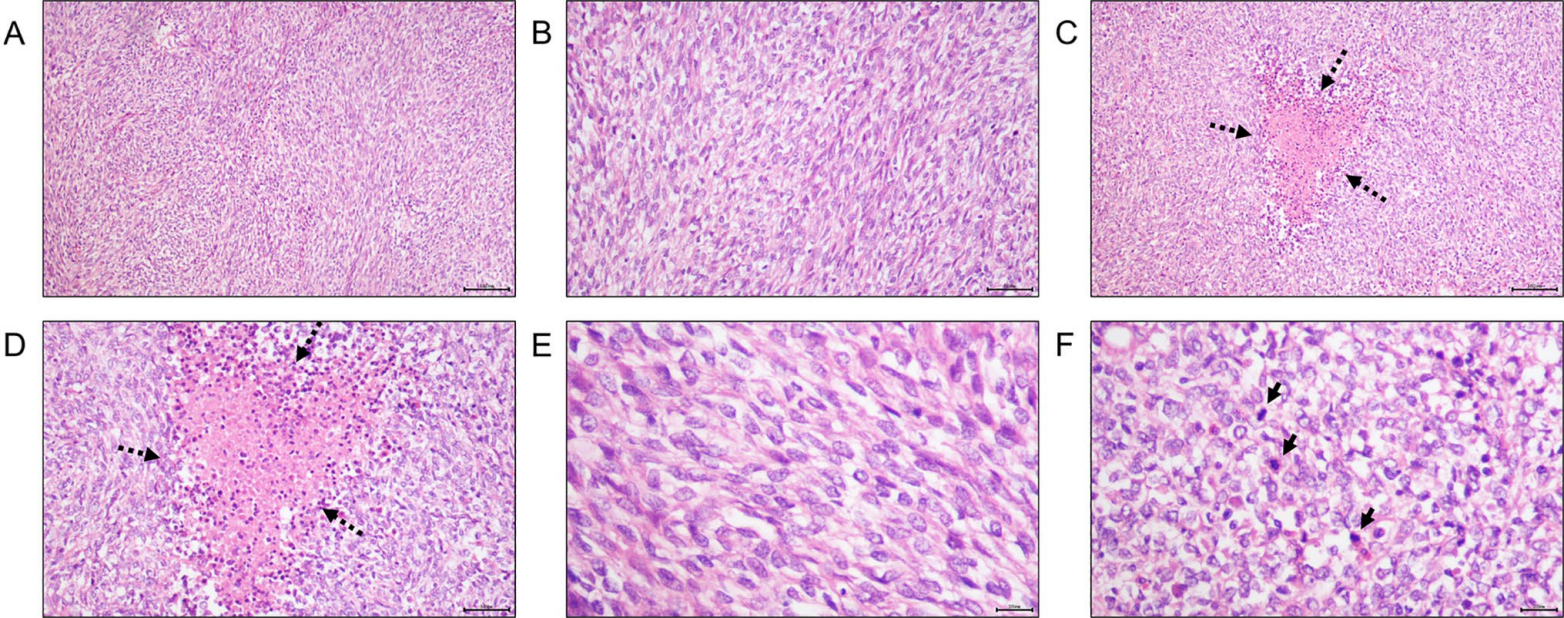
Postoperative pathology result of this case. **A** Small round blue cells with diffuse growth pattern, **B** bundles in part, **C**, **D** areas of neoplastic necrosis (the black arrow shows), **E** pale tumor cell nuclei with round, ovoid, and short spindle shape, delicate chromatin with visible nucleoli, **F** areas of pathological mitotic figures (The black arrow shows). Bar: 100X, 100 μm; 200X, 50 μm; 400X, 20 μm

**Fig. 2:**
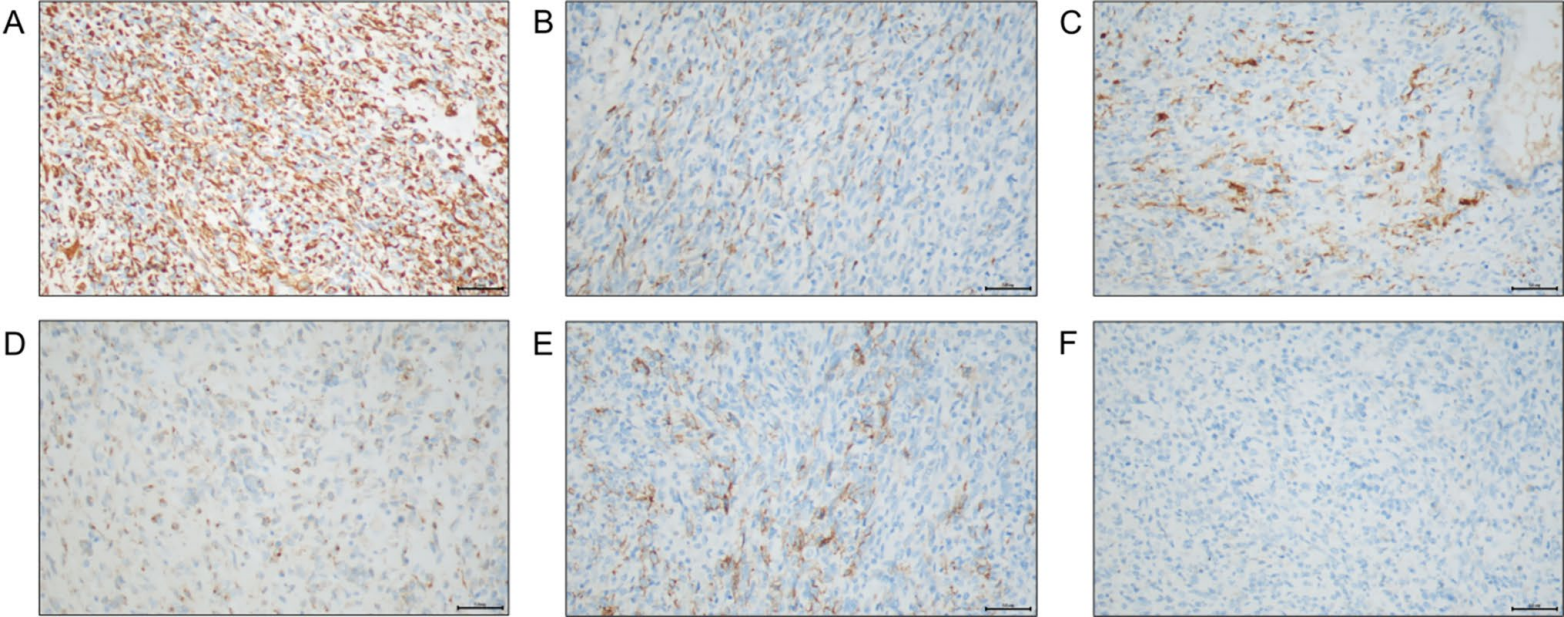
Immunohistochemistry result of this case. **A** Vimentin A (positive, diffuse), **B** CK (AE1/AE3) (positive, focal), **C** S-100 (positive, focal), **D** Syn (positive, focal), **E** CD56 (positive, focal), **F** CgA (negative). Bar: 200X, 50 μm

To further deal with dysuria, the patient was hospitalized in local hospital. Physical examination upon admission of the patient showed the prostate had increased volume with hard texture and the central groove disappeared, oppressed the rectum. No other apparently positive signs were found. He had no special medical, family, and psycho-social history except chronic B-viral hepatitis for over 30 years and denied any alcohol, drug or smoke consumption. So he underwent transurethral Holmium laser prostate surgery. Postoperative pathology result revealed small blue round cell malignant tumor (Fig. 1). To further clarify the pathological types of tumor tissue, immunohistochemistry (IHC) was performed, suggesting high-grade prostate cancer with neuroendocrine and neuroectodermal differentiation (Fig. 2). To further confirm the diagnosis, fluorescence in situ hybridization (FISH) examination was performed and found no SYT gene disruption and rearrangement, and no EWSR1/FLI1 fusion gene.

**Fig. 3:**
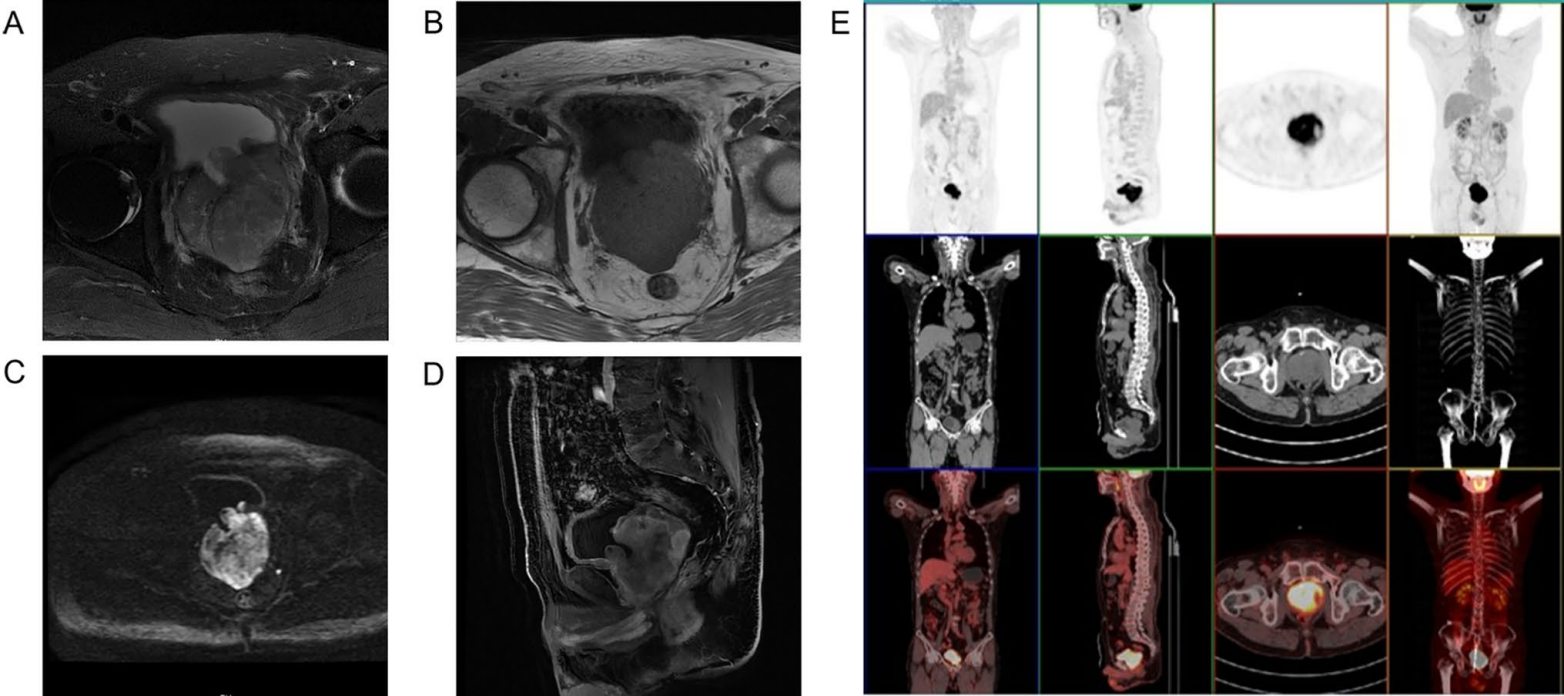
MR showed irregular tumor mass in prostate with uneven signal on T2WI image, obviously high signal on DWI image. **A** T1WI image, **B** T2WI image, **C** DWI image, **D** water image (sagittal). **E** PET–CT showed abnormal high glucose metabolism of the prostate but no distant organ metastasis was found

The dysuria symptoms significantly relieved but aggravated again 1 month after operation, and the symptoms of dribbling at the end of urination appeared, accompanied with increased frequency of urination at night (1-h interval). For further treatment, the patient presented to our hospital. The patient underwent magnetic resonance (MR) examination and found prostate enlargement with irregular masses, unclear boundary between central lobe and peripheral lobe, uneven signal on T2WI image, obviously high signal on DWI image (Fig. 3A–D). Further PET–CT found that abnormal glucose metabolism of the prostate was increased and the posterior wall of the bladder was involved but no distant organ metastasis was found (Fig. 3E). Therefore, after multiple disciplinary team discussion, a da Vinci robotic prostatectomy was performed. Tumor resection specimens were subjected to further whole-genome sequencing. The results suggested that there were three somatic variations that may have clinical significance, including *RAF1* (*CCDC6-RAF1* fusion)*, ARID1A*, and *SMARCA4*. Moreover, one germline variation that may have clinical significance is *BCL2L11* (2903-bp deletion). Besides, it was found that the tumor mutation burden was 2.33 and microsatellite stable (MSS). What is more, there were no mutation on *ALK, BRAF, BRCA1/2, PD-L1, EGFR, EGFR2/3, HER2, KIT, KRAS, MET, NRAS, NTRK1/2/3, PDGFRA, PIK3CA, RET, ROS1*, which have potential Food and Drug Administration (FDA)-approved targeted drugs for choosing. The test results did not find specific targeted drugs that could be used clinically in this patient at present.

Postoperative recovery was uneventful and the patient was discharged on the 11th postoperative day with smooth urination. Then the patient received four cycles of Ewing-type therapeutic regimens treatment (Vinorelbine Tartrate 30 mg on Day 1 + Epirubicin Hydrochloride 70 mg on Day 1–2 + cyclophosphamide 1 g on Day 1) every 3 weeks. He has been followed up to date and is currently undergoing stable follow-up for over 24 months.

## Discussion

SRCT is a group of different histologic-type neoplasms including Ewing’s sarcoma (EWS)/primitive neuroectodermal tumors (PNET), Askin’s tumor, melanocytic neuroectodermal tumor, neuroblastoma, olfactory neuroblastoma, poorly differentiated synovial sarcoma, mesenchymal chondrosarcoma, rhabdomyosarcoma, Wilms’ tumor, desmoplastic small round cells tumor and so on. In addition, tumors with small cell morphology similar to SRCT contain non-Hodgkin’s lymphoma, round cell liposarcoma, extraskeletal myxoid chondrosarcoma, poorly differentiated malignant peripheral nerve sheath tumor, malignant melanoma, rhabdoid tumor, germ cell tumors, small cell carcinoma, Merkel cell carcinoma (Meis-Kindblom et al. 1996).

In this paper, we described a rare case of prostate SRCT due to dysuria which is never reported before. Its clinical manifestations lack typicality, and ultrasound, CT, MRI with other imaging examinations are not specific. Differential diagnosis is difficult with prostate cancer and BPH, which may cause treatment delay. Pathological examination is the main diagnostic basis.

SRCT has the same morphology and size, and they are diffusely distributed in tissues with poor differentiation, high malignancy, and poor prognosis. Further histologic type is defined by IHC (Sharma et al. 2017). Besides, genetic testing is another diagnostic tool that helps SRCT be correctly classified. Most classical SRCT cytogenetic features are chromosomal aberrations, mainly translocations and proliferation of chromosome fragments (Rabbitts 1994). Studies have suggested that chromosomal translocations are primary. Soft tissue tumor-specific chromosomal aberrations lead to the recombination and fusion of protein-coding genes, which can lead to the expression of cancer-promoting proteins. The combination of the two protein-coding genes facilitates fusion gene formation, which is translated into a fusion (chimeric) protein after transcription. These two mechanisms will lead to malignant transformation of tumors or uncontrolled cell proliferation (Xia and Barr 1990). In this case, the tumor type could not be classified into current classification, but showed EWS/ PNET like.

Ewing-like SRCT comprises tumor with EWSR1 rearrangements with a non-ETS family partner and tumor with non-EWSR1-ETS rearrangements (Pappo and Dirksen 2018; Antonescu 2014). The most frequent translocations in non-EWSR1-ETS rearranged SRCT occur between the CIC gene on chromosome 19 and DUX4 on chromosomes 4 and 10, resulting in t(4;19)(q35;q13) or t(10;19)(q26;q13) (Machado et al. 2016). In addition, about 4% of non-EWSR1-ETS rearranged SRCT has BCOR rearrangements or BCOR-CCNB3 fusions (Pierron et al. 2012). Besides, there have been tumor types with NFATC2 and PATZ1 fusion. Despite significant morphological overlap, most of these subsets tend to exhibit morphological features predicting potential molecular changes. Ewing’s sarcoma is the prototype of round cell sarcoma, while in CIC sarcoma, focal polymorphism and epithelial morphology may dominate. BCOR sarcomas usually exhibit spindle-like tumor cell population. NFATC2 sarcoma may show significant epithelioid features, while PATZ1 sarcoma usually has a sclerotic background (Sbaraglia et al. 2020). The CHIP and whole-genome sequencing result in our study showed non- EWSR1-ETS rearrangements, non-EWSR1-ETS rearrangements and non-BCOR-CCNB3 fusions, indicating a new found unique subtype.

There is currently no standard treatment for SRCT. Most treatments are performed with early surgical resection and are supplemented with radiotherapy and chemotherapy. Although it has a unique genetic mutation that is different from typical Ewing sarcoma or other well-defined Ewing-like SRCT, Ewing-type therapeutic regimens was chose as a follow-up treatment for this patient (Marino-Enriquez and Fletcher 2014). According to the whole-genome sequencing results, three somatic variations may have clinical significance, including *RAF1* (*CCDC6-RAF1* fusion)*, ARID1A*, *SMARCA4*, and one germline variation *BCL2L11*. Cell experiments have shown that spontaneous activation of RAF1 can induce estrogen-independent growth of breast cancer cells and overexpression of continuously activated RAF1 protein can cause cell resistance to adriamycin and paclitaxel (El-Ashry et al. 1997; Davis et al. 2003). In addition, in vitro cell experiments showed that after the ARID1A gene was deleted, the ability of PARP inhibitors (Veliparib, Olaparib, Rucaparib, BMN673) to promote apoptosis was enhanced (Shen et al. 2015). Low ARID1A expression is associated with activation of the PI3K/mTOR signaling pathway (Huang et al. 2014). In vitro cell experiments have shown that ARID1A-deficient breast cancer cells have increased sensitivity to PI3K inhibitors (Buparlisib) and AKT inhibitors (MK-2206 and Perifosine) (Samartzis et al. 2014). In vitro cell experiments showed that ARID1A knockout lung cancer cells were sensitive to ionizing radiation, cisplatin, and UV (Watanabe et al. 2014). As for SMARCA4, in non-small cell lung cancer, SMARCA4 deletion is sensitive to combination of etoposide and DZNep (EZH2 inhibitor) (Fillmore et al. 2015). Besides, studies have confirmed that BCL2L11 deletion is associated with poor response to EGFR TKI treatment (Huang et al. 2015; Nie et al. 2015). Blocking these genes seems to be an effective treatment strategy but there are currently no drugs that specifically target. Furthermore, this case showed low tumor mutational load and MSS predicting poor immunotherapy responsiveness including PD-1/PD-L1 treatment (Rosenberg et al. 2016; Snyder et al. 2014; Rizvi et al. 2015; Le et al. 2015).

Although rare, this case highlights the need to consider malignant disease such as SRCT as a differential diagnosis for dysuria patient without elevated serum PSA level. Early detection and pathological examination is needed when there have unexpected disease progression.

## Conclusion

Prostate Ewing-like SRCT is rare and difficult to preoperatively diagnose. Even through clinical features, imageology examination, frozen pathological examination, we cannot obtain an exact diagnosis. To find unexpected prostate SRCT, any suspicious details of prostate-related symptoms or clinical diagnosed BPH should be taken into account during the follow-up of drug treatment. More importantly, new targeted drugs for the disease need to be developed for clinical application in the future.

## Data Availability

The datasets generated during and/or analyzed during the current study are available from the corresponding author on reasonable request.
